# Metacognition of curiosity: People underestimate the seductive lure of non-instrumental information

**DOI:** 10.3758/s13423-023-02404-0

**Published:** 2023-11-06

**Authors:** Sunae Kim, Michiko Sakaki, Kou Murayama

**Affiliations:** 1 Department of Psychology, Health, & Professional Development, Faculty of Health and Life Sciences, Oxford Brookes University, Gipsy Lane, Oxford, OX3 0BP UK; 2 Hector Research Institute of Education Sciences and Psychology, University of Tübingen, Tübingen, Germany; 3 Research Institute, Kochi University of Technology, Kochi, Japan; 4 School of Psychology and Clinical Language Sciences, University of Reading, Reading, UK

**Keywords:** Curiosity, Metamotivation, Information seeking, Metacognition, Affective forecasting

## Abstract

Curiosity – the desire to seek information – is fundamental for learning and performance. Studies on curiosity have shown that people are intrinsically motivated to seek information even if it does not bring an immediate tangible benefit (i.e., non-instrumental information), but little is known as to whether people have the metacognitive capability to accurately monitor their motivation for seeking information. We examined whether people can accurately predict their own non-instrumental information-seeking behavior. Across six experiments (Experiments 1A–1E and 2, total *N* = 579), participants predicted that they would engage in information-seeking behavior less frequently than they actually did, suggesting that people tend to underestimate the motivational lure of curiosity. Overall, there was no consistent statistical evidence that this underestimation was altered by contextual factors (e.g., the cost to seek information). These results were consistent with the theoretical account that it is difficult for people to make sense of the internally rewarding value of information in advance.

**Supplementary Information:**

The online version contains supplementary material available at 10.3758/s13423-023-02404-0.

## Introduction

From infancy onwards, we are curious about many things. Curiosity generally elicits exploratory behaviors (Litman et al., 2005; Loewenstein, 1994), enhances learning and memory (Fastrich et al., 2018; Gruber et al., 2014), and benefits social interaction (Kashdan et al., 2011). Curiosity is also associated with higher emotional intelligence (Leonard & Harvey, 2007), increased psychological well-being (e.g., Kashdan et al., 2004) and innovation/creativity (e.g., Hagtvedt et al., 2019). Despite its importance in our daily lives, less is known about whether people have accurate metacognition about their own curiosity. The current research aims to address this question — do we really know how curious we are?

### Curiosity as motivation to seek non-instrumental information

The definition of curiosity varies in the literature (Jirout & Klahr, 2012; Murayama et al., 2019), but this article defines curiosity as a desire to seek non-instrumental information – information that does not directly lead to immediate tangible benefits, such as money or food (Gottlieb & Oudeyer, 2018). Such a desire may be acquired phylogenetically or developmentally by the fact that information is generally useful for learning (Schmidhuber, 1991) or for making accurate predictions about the world (Friston et al., 2017). Previous literature has suggested that humans and non-human animals are inherently curious (Hayden et al., 2009; Kang et al., 2009; Kidd & Hayden, 2015; Zentall, 2016) – they are motivated to seek seemingly useless information even at the cost of their resources (e.g., delaying the time to obtain rewards).

For example, in Rodriguez Cabrero et al. (2019), participants took part in a card game on a computer. In each trial, a card type was revealed in 5 s and a certain card type was associated with obtaining rewards (points). During these 5 s, participants could choose to reveal the card type themselves by clicking a button. In some trials, participants had to pay a cost (via points deducted) in order to reveal the cards early. Importantly, revealing the cards early did not affect the trial’s outcome (i.e., the information about card type was non-instrumental). Various task characteristics, such as the level of costs (0, 5, 10, and 15 points) and the magnitude of rewards (50, 100, 300, and 500 points), were manipulated to examine their influence on information-seeking behavior. The researchers hypothesized that participants’ information seeking would be sensitive to these factors – for example, as the cost decreased the tendency for information seeking would increase. However, participants’ information seeking was dependent on cost levels but no significant impact of rewards or outcome probability on curiosity was observed (but see Golman & Loewenstein, 2018, for the effects of reward magnitude). The main finding of the study was that participants revealed cards early, even in situations where they had to pay a cost, suggesting that people attribute intrinsic value to non-instrumental information. Similar findings have been obtained in different experimental paradigms (e.g., Bennett et al., 2016; Charpentier et al., 2018; FitzGibbon et al., 2021), involving non-human animals (e.g., Bromberg-Martin & Hikosaka, 2011; Hayden et al., 2009), and different types of costs (e.g., information obtained at the cost of waiting time; van Lieshout et al., 2018; see also Marvin & Shohamy, 2016).

Many researchers have explained these findings by assuming that information has intrinsic value – information is considered rewarding by itself (Gottlieb & Oudeyer, 2018; Gruber & Ranganath, 2019; Kang et al., 2009; van Lieshout et al., 2018). FitzGibbon et al. (2020) further proposed that information possesses a so-called incentive salience property (see also FitzGibbon & Murayama, 2022; Litman, 2005). Incentive salience refers to the motivational feeling or urge of “wanting” that directs people’s behavior even without accompanying subjective positive feelings (i.e., “liking”). The concept of incentive salience was originally proposed to explain impulsive behaviors related to extrinsic incentives such as drug addiction (see Berridge, 2012; Robinson & Berridge, 2008) but it can additionally be used to explain seemingly irrational seeking behavior for non-instrumental information observed in the aforementioned studies. Supporting this idea, Lau et al. (2020) showed that people are willing to run the risk of receiving an electric shock in order to satisfy their curiosity and hunger for foods, and the effects were related to the activation in the dorsal striatum, one of the brain areas related to incentive salience (Lawrence et al., 2012).

### Metamotivation of curiosity

Metacognition is defined as the ability to monitor and control our cognitive states and processes (e.g., Koriat, 2007; Nelson & Narens, 1990). We may think that we know ourselves very well, but previous work has suggested that we are often quite inaccurate in predicting our future behaviors, learning, motivation, preferences, and attitudes (e.g., Bjork et al., 2013; Kahneman & Snell, 1992; Loewenstein et al., 2003; Loewenstein & Schkade, 1999; Wilson & Gilbert, 2003). The current study focuses on a form of metacognition called *metamotivation*: the ability to monitor and control our motivational states (Murayama, 2014; Scholer et al., 2018). Accurate beliefs and assessments about one’s own motivation are important because they allow us to adopt effective strategies that enhance performance and learning (Murayama et al., 2016). To illustrate this point, imagine that you misjudge your internal motivation to undertake a certain task and inaccurately believe that external incentives help you to complete it. The decisions based on such inaccurate metamotivation (e.g., not engaging in the task, setting up external incentives) would hinder your performance and even further decrease your internal motivation. However, prior studies show that people’s metacognitive accuracy of motivation is sometimes biased (Murayama et al., 2016; Scholer & Miele, 2016), suggesting that their metacognitive accuracy of the motivational property in curiosity may also be imperfect.

Murayama (2022) recently proposed a reward-learning framework of knowledge acquisition, which predicts that people cannot fully appreciate the intrinsic value of information. In other words, people tend to *underestimate* the motivational lure of curiosity. This underestimation occurs because (1) information is intangible by itself, making it difficult to appreciate its value until one obtains it, and (2) the process by which information acquires rewarding value is complex and people are unable to track it. Several studies have provided partial evidence for this claim. For example, Kuratomi et al. (2022) showed that participants rated their enjoyment and engagement levels for a boring cognitive task (e.g., flanker task) higher than they initially expected when there were no performance-contingent incentives, thus demonstrating that people underestimate the task motivation (see also Hatano et al., 2022). In addition, the underestimation effect was significantly diminished when participants were provided with performance-contingent rewards, indicating that non-instrumental aspects of the task were responsible for the observed effect (see also Heath, 1999; Woolley & Fishbach, 2015).

However, previous research does not directly address curiosity itself, i.e., spontaneous seeking of non-instrumental information. The boring task used by Kuratomi et al. (2022) does not have any explicit information-seeking aspect. Furthermore, there is a critical difference between the boring tasks and information-seeking tasks typically used in research on curiosity: the perceived reward value or valence. People generally perceive boredom as negative and associate it with low rewarding values (Pekrun, 2006). Therefore, it is intuitive that individuals may underestimate their enjoyment for upcoming boring tasks due to such a general negative perception. In contrast, people may have a positive view of seeking non-instrumental information – which is inherently rewarding (Gruber & Ranganath, 2019; Kang et al., 2009; Lau et al., 2020). To examine the metacognitive accuracy of curiosity we should utilize a task that directly tests the positively rewarding nature of information.

To our knowledge, the only study that directly addressed the metacognitive accuracy of non-instrumental information-seeking behavior is the unpublished work by Loewenstein et al. (1998). In this study, participants were assigned to one of two conditions. In the “actual” condition, they first answered trivia questions and had to choose between two options: (1) seeing correct answers to the questions or (2) receiving a candy bar (in which case they did not see the correct answers). Finally, they were asked to rate their current/actual level of curiosity on a scale of 1 to 10. In the “predictive” condition, participants were first provided with a sample question with a correct answer and were asked to predict which option they would choose: (1) seeing the correct answers to the rest of the questions or (2) receiving a candy bar. They also predicted their future curiosity level if they were to try to answer similar questions. Participants in the actual condition were more likely to choose to see the correct answers than those in the predictive condition had predicted. The findings are therefore consistent with the idea that people tend to underestimate the motivational property of curiosity.

While Loewenstein et al. (1998) conducted an initial study that tested whether adults underestimate their future curiosity (although unpublished), considering the scarcity of evidence in the literature, additional studies would strengthen the generalizability of the finding. Additionally, in their study, participants made a prediction after their curiosity was already satiated (i.e., the answer was provided). It is possible that this satiation process prompted participants to underestimate the motivational lure of curiosity. Moreover, given that participants in the predictive condition did not see actual trivia questions, they may have imagined very different trivia questions from those presented in the other condition. Finally, in light of the curiosity literature discussed earlier (e.g., Rodriguez Cabrero et al., 2019), adults’ underestimation of future curiosity may be influenced by other factors such as the level of information-seeking costs.

### Current study

We aimed to examine the metacognitive accuracy of the motivational property of curiosity. We specifically tested the hypothesis that people tend to underestimate the motivational property of curiosity (Murayama, 2022). To directly address non-instrumental information-seeking behavior, we adopted the paradigm used by Rodriguez Cabrero et al. (2019). In this paradigm, participants engaged in a card game where the identity of the card was revealed in 5 s. The card’s identity was probabilistically determined and determined the monetary rewards. Importantly, during the 5 s participants had the option to reveal the identity earlier by incurring some costs (points deducted). The information about the card’s identity was non-instrumental (i.e., seeing the information would not change the card’s identity and participants could gain any advantage from knowing the information). We compared the actual frequency of information-seeking behavior with the frequency that participants had predicted beforehand. To align with previous paradigms on curiosity (e.g., Rodriguez Cabrero et al., 2019), we also varied the levels of rewards (points associated with a certain card identity), costs (associated with revealing cards early), and the probability of obtaining a certain card identity. We conducted an exploratory analysis to examine the potential impact of these factors on metacognitive accuracy. Although Rodriguez Cabrero et al. (2019) found little impact of these factors on the information-seeking behavior itself (except for the cost), we considered that they might have an impact on meta-cognitive judgments in a different manner, given the number of studies showing that meta-cognitive judgments are often selectively influenced by certain factors (e.g., Jacoby et al., 1989; Rhodes & Castel, 2008). We expected that people would generally underestimate the motivational property of curiosity: People’s predicted frequency of information-seeking behavior would be less than the actual information-seeking frequency.

## Experiments 1A–1E

### Method

To test our hypothesis, we conducted five experiments (Experiments 1A–1E) that differed in minor aspects of the task. Because all experiments were similar, we summarize them together rather than reporting them separately.

#### Participants

A total of 243 participants (137 females, 106 males) were recruited through Prolific (https://www.prolific.co/). Participant information for each experiment including ethnic background and educational attainment can be found in the Online Supplementary Materials (OSM). We predetermined the sample size for Experiment 1A based on the available budget at the time and a power analysis (we planned to recruit 50 participants for the first experiment, which was deemed sufficient to detect Cohen’s *dz* = .40 at a power of 80%). As the first experiment supported the hypothesis, we decided to collect a similar sample size for the following experiments (1B–1E). For transparency, our decision to summarize the data from the five experiments was rather ad hoc due to some unexpected interaction effects that appeared only in one or a few experiments. However, no optimal stopping rules were applied to data collection and we report all the similar experiments we conducted (i.e., no publication bias); therefore, the effect size is unlikely to be overestimated (Ueno et al., 2016). A sensitivity power analysis^1^ showed that a sample size of 243 in an experiment is sufficient to detect an effect size of Cohen’s *dz* = .18 at a power of 80%. This sensitivity analysis was conducted on a paired-samples *t* test for convenience as our main focus was on the paired comparison between the predicted and actual behavior, although we used ANOVA to account for other factors. The study was approved by the University Research Ethics Committee at the University of Reading (ethics number: 2016-109-KM).

#### Common procedure

The procedure was similar across five experiments that were administered via an online platform, Gorilla (https://gorilla.sc/). The main task was an adapted version of the task used by Rodriguez Cabrero et al. (2019); participants played a card game in which they saw a card and won either 50 or 100 points if the flipped card included a certain symbol (i.e., heart). The chance of revealing the heart was varied (25%, 50 %, or 75%) within participants.

In each trial, participants saw a card placed face-down with a question mark visible on the back side of the card, and after 5 s, the card was flipped to reveal its identity. The countdown timer was located in the upper right corner of the screen. During the 5 s, participants were allowed to flip the cards early by clicking the *reveal* button. However, revealing the cards incurred a cost of 0, 10, or 20 points. That is, if participants chose to reveal the card early, they had to pay for these points (when the cost was 0, participants could reveal the card early without any cost). In addition, revealing the card did not affect the length of the trial (i.e., the identity of the card was displayed longer when participants revealed the card early). This was important to prevent participants from revealing the card early simply to finish the experiment sooner. When the card revealed its identity, the screen also displayed the accumulated earned points.

**Fig. 1:**
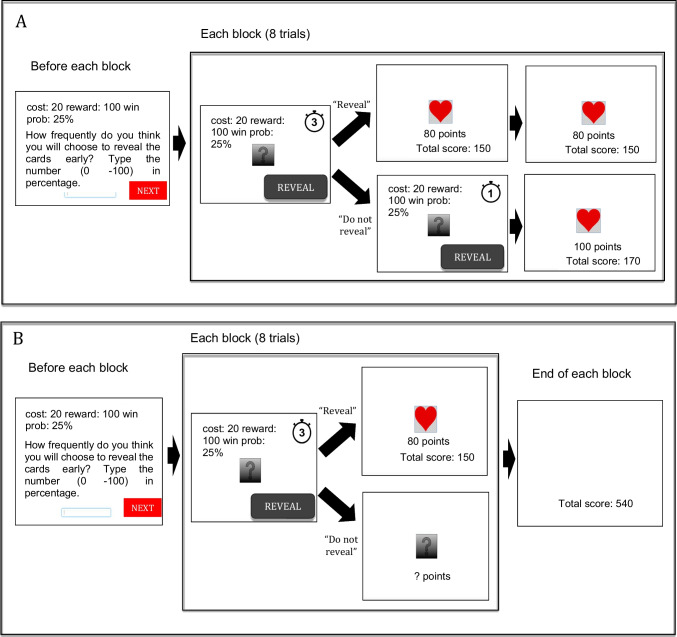
Participants were first asked to predict how frequently they would reveal cards in the beginning of each block. In each trial, participants could reveal cards within 5 s and the outcomes were revealed regardless of whether they revealed the cards in Experiments 1A–1C (**A**: an upper panel). The outcomes were revealed only when they paid costs but at the end of each block participants saw the total earned scores regardless of whether they paid costs in Experiments 1D and 1E (**B**: a lower panel)

Participants performed a total of 18 blocks, each of which corresponded to one of the 18 conditions (two reward levels: 50 points, 100 points; three cost levels: 0 points, -10 points, -20 points; three win-probability: 25%, 50 %, 75%; all of them were factorially combined). In Experiment 1C, only two cost level conditions (0 points and -10 points) were included (see OSM). Each block consisted of eight trials. The order of the blocks was completely randomized, as was the order of outcomes across trials (within a block). Importantly, prior to each block, participants were informed of the cost, reward, and win probability for the upcoming block. They were then asked to make a prediction about how frequently they would reveal the cards early in that block (via a percentage). See Fig. 1 for a schematic representation of the procedure.

Before the task, participants read instructions about the card game. Most importantly, they were explicitly instructed that revealing cards early would not change the total duration of each trial (see OSM). Similar to Rodriguez Cabrero et al. (2019), participants viewed an increase or decrease in points (not in money) during the game and received monetary rewards at the end. However, participants in our study were clearly instructed that their bonus payment would be based on their total scores, although the conversion rate of points to actual payment was not specified in advance. This meant that participants interpreted the cost relative to the reward points they could potentially gain. In fact, at the end of the study after receiving an initial payment as indicated in an advertisement of the study in Prolific, all participants received the same additional small payment. As will be seen, there was a robust main effect of cost and reward manipulation in all experiments, suggesting that participants understood the value of these reward points well. Right after the instructions, participants completed a comprehension check question asking whether choosing to reveal the card would end the task early or not. Finally, participants received two practice trials prior to the main task.

At the end of the study, participants received three attention check questions. Prior to data analysis, we excluded data based on participants’ responses to the attention check questions (see OSM for the details of these questions and excluded *N*s).

#### Procedural differences of Experiments 1A–1E

The basic structure of the five experiments was the same, but they were different in the following respects. First, as mentioned earlier, we removed the cost -20 condition in Experiment 1C due to a floor effect. Second, in Experiments 1B–1E, participants received feedback on whether their response regarding the task duration was correct or not before starting the main task, while in Experiment 1A the same question was also asked without feedback right after the task. In addition, in Experiments 1B–1E, a summary of the game was presented right before the main task, whereas in Experiment 1A, the game instructions were presented across several pages without a summary at the end. These changes were made to ensure participants’ understanding of the task. Furthermore, in Experiments 1A–1C, even if participants decided not to see the identity of the cards in advance (including when costs were involved to view the identity), the card identity was revealed right at the end of the trial (Fig. 1). In other words, the decision to view the identity early did not provide much informational value. In Experiments 1D and 1E, in contrast, participants were able to view the card identity in each trial only if they paid the associated costs. Moreover, while participants were asked to predict the frequency of their own card reveals in percentage in Experiments 1A–1D, they were asked to predict the frequency of another person (similar to them) revealing the card in Experiment 1E. This procedure was adopted from previous studies on affective forecasting (e.g., Igou, 2008) and on metacognitive accuracy (e.g., Kassam et al., 2009), and we wanted to test the robustness of the results using a different method of assessing metacognition. Finally, we included the Intolerance of Uncertainty questionnaire at the end of the study in Experiments 1A and 1B, but not in Experiments 1C–1E. This questionnaire was included for exploratory purposes only (see OSM for the results). Materials used for all the experiments are available at: https://app.gorilla.sc/openmaterials/466834.

### Results

#### Analysis of individual experiments

We analyzed the data from individual experiments using 2 × 3 × 2 × 3 ANOVA including Prediction (predicted and actual percentage), Cost (0, 10, and 20 points), Reward (50 and 100 points), and Win probability (25%, 50%, and 75%) as within-subjects factors (in Experiment 1C Cost had only two levels, 0 and -10). We expected a main effect of Prediction, showing that participants predicted the frequency of reveal more than the actual reveal.

**Fig. 2:**
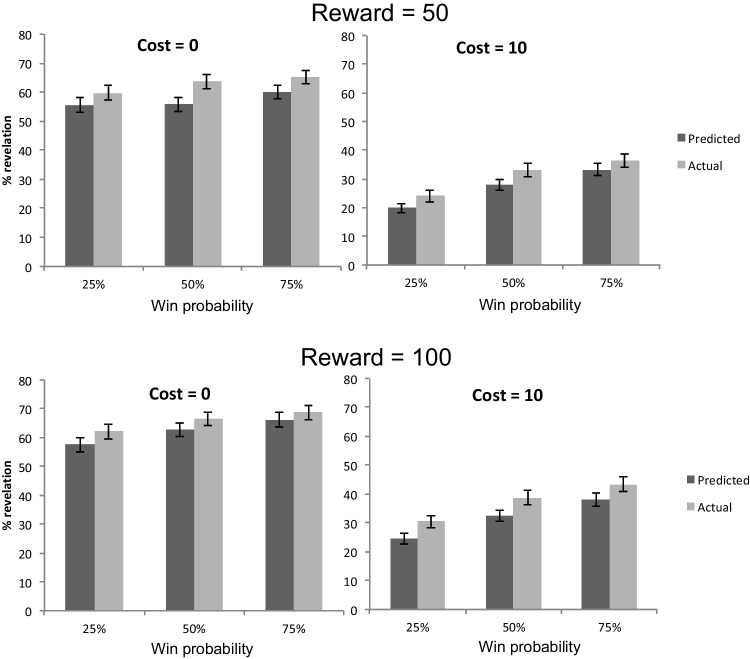
Participants’ prediction and actual revealing in percentage in five experiments. Standard errors are calculated based on SD divided by the square root of *N* within the cell

Results from individual ANOVAs are reported in the OSM. Generally, the results supported the hypothesis: the analysis showed a significant main effect of Prediction, except for Experiment 1C (the direction of the effect was the same). We also observed several significant interaction effects in individual experiments but they were not consistent across experiments (see OSM for further analyses of these individual interaction effects). Figure 2 presents the averaged results across all experiments to give a rough picture of overall pattern (note the -20 points condition in Cost was eliminated because not all experiments included that condition). We also include values of mean and standard deviation for each condition (separately for different experiments) in the OSM (see S-Table 1, OSM).

#### Integrated analysis across Experiments 1A–1E

To statistically evaluate the robustness of the effects across all five experiments, we conducted an internal meta-analysis using a linear mixed-effects model. This analysis incorporated the combined data from experiments (1A–1E), treating both participants and experiments as random effects. Thus, the results below take into account the potential heterogeneity of the experimental results. This is essentially equivalent to a random-effects meta-analysis when all data points are available. For the analysis, we excluded the cost -20 points condition as it was missing in Experiment 1C. The tested model included all possible 15 fixed-effects, including the main effects of Prediction, Cost, Reward, and Win Probability, as well as all possible two-way, three-way, and four-way interactions (a total of 11 interactions). In addition to the random intercepts of participants and experiments, we included all random slopes of participants and experiments except for the four-way interaction. The exclusion of the four-way interaction was necessary as it would have been confounded with errors, making it impossible to disentangle individual differences from measurement errors, as each participant had only one value for each combination of conditions. We employed SAS proc mixed for the analysis (Wells, 2021) as it computes the statistical significance of omnibus effects using a general linear model framework, which is not available in the other alternatives (e.g., lme4 package in R). We adjusted degrees of freedom and standard errors to account for small sample sizes using the Kenward-Roger method (Kenward & Roger, 1997).

As expected, the full model generated error messages due to negative variances in several random effects, indicating that the model was overly complex. We fixed these negative variances to zero and re-analyzed the data using the same mixed-effects model analysis with reduced random effects components. Note that even though the random slopes of experiments for Prediction were estimated to be zero in the initial analysis, we still included them in the subsequent analysis because Prediction was of utmost interest. The revised model converged successfully and the results are summarized in Table 1. To assess the statistical significance of the random effect variances, we conducted a log-likelihood ratio test by comparing the model with a particular random effect to the model without that random effect.

**Table 1:** Integrated analysis results with mixed-effects modelling

Effect	DF (numerator)	DF (denominator)	*F* value	*p* value	Random effect variance of Subject	Random effect variance of Exp
Prediction	1	11.6	11.94	.005	55.387**	0
Cost	1	4.02	63.02	.001	315.29**	18.324**
Reward	1	8.25	7.34	.026	7.538	–
Win Probability	2	20.4	18.23	<.001	23.663**	–
Prediction × Cost	1	6.55	.46	.520	18.244**	4.378
Prediction × Reward	1	8.18	.58	.468	–	–
Prediction × Win Probability	2	9.96	.93	.365	–	1.930
Cost × Reward	1	7.72	.93	.365	25.996**	1.502
Cost × Win Probability	2	12.6	6.81	.001	33.458**	2.437
Reward × Win Probability	2	16.1	1.31	.298	–	–
Prediction × Cost × Reward	1	8.18	.18	.681	–	8.604**
Prediction × Cost × Win Probability	2	2541	2	.136	–	–
Prediction × Reward × Win Probability	2	2542	.14	.866	–	–
Cost × Reward × Win Probability	2	161	1.31	.298	106.86**	3.087
Prediction × Cost × Reward × Win Probability	2	2541	2.83	.059	–	–

– means that these random effects were not estimated. For random effects variance, * *p* < .05, ** *p* < .01

Consistent with our hypothesis, the fixed-effect of Prediction was statistically significant, *F* (1, 11.6) = 11.94, *p* = .005, suggesting that the underestimation effect is generalizable across experiments (note that we conducted a random-effects meta-analysis). The estimated beta coefficient for this effect was 6.58, meaning that on average participants’ predictions were 6.58% lower than their actual card-revealing behavior. The random slopes of the experiment were estimated to be zero, suggesting no evidence of heterogeneity in the effect across experiments.

Importantly, none of the interaction effects involving Prediction reached statistical significance (Table 1). This indicates that the significant interaction effects observed in individual experiments were not robust and did not provide strong evidence for differential effects of factors such as cost, win probability, and reward on metacognitive prediction and actual information-seeking behavior. However, we observed significant between-experiment variance for the effects related to Prediction (i.e., Prediction × Cost × Reward), suggesting the possibility that the pattern of results for this interaction may differ across experiments. We describe and discuss this interaction effect in the OSM.

## Experiment 2

In Experiments 1A–1E, participants were asked to make predictions prior to information seeking in each block. With this design, there is a possibility that the act of prediction influenced subsequent information-seeking behavior (e.g., participants may have attempted to align their information seeking with their predictions). To examine whether making predictions influenced actual information-seeking behavior, we conducted a large-sample experiment in which we compared two groups of participants. Specifically, one group took part in the card-revealing game following the same procedure as in the previous Experiments 1A–1E (i.e., the prediction group), while the other group participated in the card revealing game without making a prior prediction (i.e., the no-prediction group).

### Method

#### Participants and procedures

We conducted only one specific block (although the total number of blocks was not informed to participants), which previously demonstrated the largest effect size (Cost-10, Reward-100, Win probability-50 condition in Experiment 1D). Based on the minimum effect size of interest *d* = .3, an alpha level of .05, and 80% power, we obtained the desired sample size of 352 participants. After applying the same exclusion criteria, the final sample consisted of 336 participants (194 males). Detailed information about the participants can be found in the OSM.

### Results

**Fig. 3:**
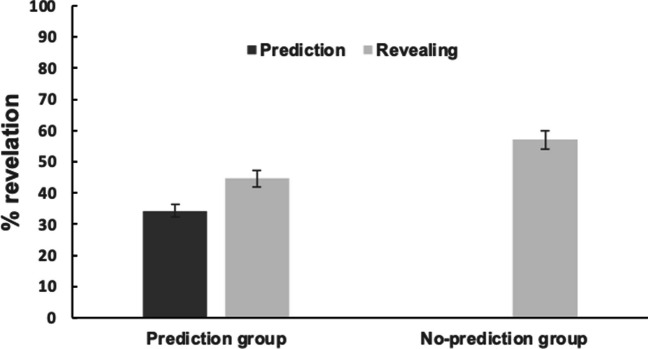
The prediction and actual card-revealing in percentage in the prediction group and actual card revealing in the no-prediction group in Experiment 2. The no-prediction group does not have a predicted percentage because participants did not make a prediction

Figure 3 presents the results. In the prediction group, replicating our main findings, participants underestimated their information-seeking behavior by revealing cards more frequently than they had initially predicted *t* (170) = -5.35, *p* < .001, *d* = -.41. Importantly, we also observed a significant difference in information-seeking behavior between the two groups, *t* (334) = 3.14, *p* = .002, *d* = .34. Interestingly, participants in the no-prediction group revealed cards *more frequently* compared to those in the prediction group. As a result, when comparing the predicted information-seeking behavior in the prediction group and actual information-seeking behavior in the no-prediction group, the underestimation was larger, *t* (334) = -6.30, *p* < .001, *d* = -3.60. This suggests that the act of prediction might have influenced people’s actual information-seeking behavior in previous experiments – but in a way that has *reduced* the observed underestimation effect. These findings strengthen our claim that individuals tend to underestimate their own information-seeking tendencies.

## General discussion

In the present research, we tested the hypothesis that people tend to underestimate the motivational property of curiosity. To examine this, we utilized a well-established paradigm from previous studies (e.g., Rodriguez Cabrero et al., 2019), and compared participants’ predicted and actual non-instrumental information-seeking behavior. The results supported the underestimation hypothesis, revealing that participants’ predicted frequency of seeking non-instrumental information was lower than their actual frequency. These findings are consistent with the theoretical account of Murayama (2022) and Loewenstein et al. (1998): it is difficult for people to anticipate the rewarding experience associated with intangible information in advance.

It is worth noting that we employed a within-subjects design for the task, allowing participants to repeatedly experience the urge of curiosity. Unlike studies on affective forecasting in which participants typically engage in a single task and rate their predicted and actual emotions before and afterwards, participants in our study had numerous opportunities to experience their actual curiosity and make accurate predictions about their information seeking tendencies in subsequent blocks. Despite this repeated exposure to the curious state and its consequences, participants continued to underestimate the motivational property of curiosity. These observations may appear puzzling but are consistent with previous findings showing the robustness of metacognitive illusions (e.g., Bjork et al., 2013; Kahneman et al., 1993; Robinson & Clore, 2002; Yan et al., 2016). Studies also showed that adults’ mis-prediction in affective forecasting is not easily corrected by experiences or feedback (e.g., Ubel et al., 2001; Walsh, & Ayton, 2009; Wilson et al., 2001), although affective forecasting tends to improve with age (e.g., Nielsen et al., 2008; Scheibe et al., 2011). It would be interesting to examine whether the underestimation effect on metacognitive accuracy in information-seeking behavior can be reduced through training or intervention (e.g., giving external feedback).

Our findings present an interesting contrast to the general findings in the fields of metacognition or affective forecasting, which typically show a tendency for people to overestimate their mental states. For example, a large body of research has provided evidence that individuals frequently predict their emotional reactions to last longer and be more intense than they actually are (e.g., Gilbert et al., 1998; Wilson & Gilbert, 2003; Wilson et al., 2000). Dunn et al. (2003) demonstrated such an overestimation of emotional intensity in a study examining students’ satisfaction with their assigned dormitory. Likewise, in the metacognition literature, research generally reports a pattern of overconfidence (i.e., overestimation of memory and learning abilities) (e.g., Kruger & Dunning, 1999; Metcalfe, 1998), although this can be modulated by factors such as expertise (e.g., Dunning, 2011).

While the exact mechanisms underlying affective forecasting or metacognition of learning are likely to be different from those underlying metamotivation, we can speculate on a common factor that could explain the different findings: saliency. Affective forecasting studies typically focus on emotional states primarily caused by visible and salient external events and which are thus easily comprehensible (e.g., happiness after winning a football game). The salience of these emotional events can be a critical reason for the overestimation observed in affective forecasting: people tend to overestimate the impact of emotionally salient events likely due to their attention and thought process being focused on them (focalism; see Wilson et al., 2000). Similarly, research on metacognition and learning suggests that individuals tend to be overconfident when dealing with visually or mentally salient materials (e.g., Rhodes & Castel, 2008). In contrast, curiosity is an emotion influenced by less salient epistemic factors such as one’s internal knowledge state (Murayama, 2022). As a result, people may have inherent difficulty in identifying and understanding their curiosity, leading to an underestimation of its motivational property.

Although the current findings align with the theoretical predictions, one critical concern is their potential generalizability. The current study employed a single paradigm that focused on an arbitrary situation. Therefore, it is unclear whether the current findings would be consistently observed in other information-seeking tasks or in our daily naturalistic behavior. Additionally, the finding that participants without a prior prediction revealed cards more frequently than those who made a prediction first in Experiment 2 suggests that the underestimation of curiosity may actually be greater than what was observed in Experiment 1. Moreover, altering the temporal distance for curiosity to be satisfied (Noordewier & van Dijk, 2017) could potentially yield different results. It is worth noting that, while no significant interaction effects related to Prediction were found across the experiments, there was a significant random effect regarding the Prediction × Cost × Reward interaction (see the OSM for a detailed discussion). This suggests that in certain situations, the cost to seek information and the rewarding value associated with the information may act as moderating factors for the observed effect. Finally, the conversion rate of obtained points into total payment was not disclosed to participants upfront. Although participants were aware that their payment would be based on their points, the presence of ambiguity may have amplified the influence of curiosity in response to the reward or cost manipulation. Building upon our findings, future studies could further explore the boundary conditions of this phenomenon in a more systematic manner.

### Supplementary Information

Below is the link to the electronic supplementary material.

Supplementary file1 (DOCX 795 KB)

## Data Availability

The data for all experiments can be found at: https://osf.io/unmfh/?view_only=3f456309347f4808a2a3ee6ed712dbc6 Materials for all experiments are also available at: https://app.gorilla.sc/openmaterials/466834 None of the experiments were preregistered.

## Footnotes

1 Please note that the sensitivity power analysis never used the effect size estimates from the experiments as input; Therefore, this is different from a post hoc power analysis (e.g., Perugini et al., 2018).
