# Supplementary material for: Metacognition of curiosity: People underestimate the seductive lure of non-instrumental information
Source: Psychon Bull Rev. 2023 Nov 6;31(3):1–12. doi: 10.3758/s13423-023-02404-0 (PMC11192831; doi:10.3758/s13423-023-02404-0)
Supplement: Supplementary file 1 — Supplementary file1 (DOCX 795 KB) [file 13423_2023_2404_MOESM1_ESM.docx]

**Supplementary Materials**

Table 1. Means and Standard deviations (in parenthesis).

|  | | | Experiment 1A | | | |  | Experiment 1B | | |  | Experiment 1C | |  | Experiment 1D | | |  | Experiment 1E | | |
| --- | --- | --- | --- | --- | --- | --- | --- | --- | --- | --- | --- | --- | --- | --- | --- | --- | --- | --- | --- | --- | --- |
| Reward = 50 | | | Cost | | | | | | | | | | | | | | | | | | |
|  |  |  | 0 | 10 | 20 |  | | 0 | 10 | 20 |  | 0 | 10 |  | 0 | 10 | 20 |  | 0 | 10 | 20 |
| 25% | Predict | 50.40 (41.8) | | 18.16 (26.2) | 13.80 (21.6) |  | | 53.02 (45.1) | 12.40 (21.8) | 16.13 (28.9) |  | 34.88 (35.7) | 21.46 (30.1) |  | 70.61 (38.1) | 17.14 (21.4) | 15.08 (23.4) |  | 69.16 (34.2) | 29.65 (27.0) | 23.06 (22.4) |
|  | Actual | 54.50  (43.4) | | 22.25 (33.2) | 16.25 (30.3) |  | | 57.22 (44.6) | 19.17 (32.8) | 18.33 (32.3) |  | 41.00 (39.8) | 21.75 (32.2) |  | 74.23 (33.1) | 26.02 (30.9) | 20.92 (28.6) |  | 72.45 (34.0) | 30.87 (31.7) | 21.43 (23.1) |
| 50% | Predict | 52.20 (38.4) | | 21.49  (27.9) | 22.40 (28.5) |  | | 41.13 (40.2) | 24.69 (31.8) | 16.73 (28.3) |  | 43.32 (35.3) | 23.58 (29.9) |  | 68.64 (35.6) | 26.12 (27.8) | 21.37 (27.3) |  | 72.39 (30.8) | 44.20 (28.4) | 27.37 (21.7) |
|  | Actual | 57.00 (37.4) | | 27.50  (35.5) | 21.50 (32.7) |  | | 57.50 (44.2) | 28.06 (35.4) | 19.17 (31.9) |  | 51.75 (39.0) | 29.00 (35.7) |  | 78.06 (33.6) | 30.36 (32.2) | 31.63 (32.4) |  | 73.98 (32.5) | 50.26 (35.8) | 31.63 (29.1) |
| 75% | Predict | 61.86 (38.6) | | 31.97 (34.2) | 21.72 (30.4) |  | | 51.44 (43.3) | 22.56 (31.0) | 23.13 (31.2) |  | 46.00 (36.7) | 22.80 (29.1) |  | 69.80 (37.2) | 36.37 (33.3) | 28.67 (32.2) |  | 70.84 (32.9) | 51.82 (35.1) | 38.41 (30.7) |
|  | Actual | 63.25 (37.5) | | 29.25 (33.5) | 19.75 (31.7) |  | | 60.00 (41.9) | 26.39 (36.4) | 25.83 (36.5) |  | 51.00 (37.5) | 25.00 (36.2) |  | 77.55 (33.0) | 43.88 (38.5) | 33.67 (34.7) |  | 73.98 (33.7) | 56.38 (39.4) | 41.07 (37.7) |
| Reward = 100 | | Cost | | | | | | | | | | | | | | | | | | | |
|  |  | 0 | | 10 | 20 | |  | 0 | 10 | 20 |  | 0 | 10 |  | 0 | 10 | 20 |  | 0 | 10 | 20 |
| 25% | Predict | 51.80 (39.4) | | 18.40 (26.9) | 14.82 (23.2) | |  | 41.38 (44.3) | 15.40 (25.1) | 15.00 (25.5) |  | 45.30 (36.5) | 25.60 (30.9) |  | 72.84 (36.3) | 27.49 (31.0) | 17.69 (26.5) |  | 75.71 (31.8) | 34.29 (25.6) | 26.41 (26.0) |
|  | Actual | 55.50 (39.7) | | 34.50 (37.8) | 19.5 (33.0) | |  | 50.83 (45.6) | 23.06 (35.0) | 22.50 (35.0) |  | 49.50 (40.2) | 21.50 (32.3) |  | 77.55 (35.0) | 29.08 (29.1) | 24.74 (30.0) |  | 76.53 (32.3) | 43.62 (34.3) | 34.69 (32.5) |
| 50% | Predict | 51.98 (39.3) | | 28.16 (29.6) | 20.46 (28.7) | |  | 57.71  (41.4) | 23.78 (33.2) | 17.91 (26.7) |  | 50.68 (38.1) | 28.70 (30.3) |  | 78.37 (33.5) | 29.90 (27.9) | 26.02 (27.4) |  | 74.69 (30.6) | 51.43 (30.3) | 40.10 (31.8) |
|  | Actual | 56.25 (38.0) | | 37.25 (37.1) | 22.00 (32.6) | |  | 63.33 (40.5) | 26.94 (38.2) | 25.83 (34.7) |  | 56.75 (39.0) | 29.50 (34.6) |  | 78.06 (32.8) | 42.35 (36.6) | 36.22 (34.1) |  | 77.55 (32.6) | 56.89 (37.3) | 43.62 (37.8) |
| 75% | Predict | 58.38 (40.7) | | 34.10 (35.1) | 27.68 (31.0) | |  | 60.73 (43.6) | 27.16 (35.0) | 25.49 (34.2) |  | 52.50 (39.2) | 29.70 (33.4) |  | 76.12 (34.3) | 42.04 (36.1) | 32.45 (33.1) |  | 83.00 (24.7) | 55.98 (36.7) | 44.96  (36.9) |
|  | Actual | 63.25 (40.8) | | 46 00  (40.7) | 25.25 (33.6) | |  | 65.83 (43.2) | 33.33 (40.1) | 29.72 (40.5) |  | 56.50 (39.1) | 33.50 (38.4) |  | 78.06 (32.7) | 45.66 (39.2) | 40.56 (37.1) |  | 79.85 (30.2) | 57.14 (39.4) | 52.55 (41.1) |

**Experiment 1A**

**Method**

*Participants.* Table 2 presents participant information for each experiment 1A-1E. Two participants’ ages were missing because they inaccurately indicated the year of testing as the year of their date of birth. An additional participant was tested but excluded based on their response to the attention check question (see the procedure below).

Table 2. Participant information for Experiment 1A-1E

|  |  | Experiment 1A | Experiment 1B | Experiment 1C | Experiment 1D | Experiment  1E |
| --- | --- | --- | --- | --- | --- | --- |
|  | Total *N* (female, male) | 50 (21, 29) | 45 (33, 12) | 50 (23, 27) | 49 (28, 21) | 49 (32, 17) |
|  | Mean age | 29 | 28.67 | 28.65 | 34.45 | 37.51 |
| Age range | | 18 ~ 65 | 18 ~ 52 | 18 ~ 55 | 18 ~ 77 | 19 ~ 68 |
| Ethnicity (*N*) | | | | | | |
|  | White | 39 | 34 | 24 | 35 | 43 |
|  | Asian | 6 | 7 | 0 | 4 | 3 |
|  | Black | 2 | 1 | 17 | 5 | 3 |
|  | Mixed/Other | 3 | 3 | 9 | 5 | 0 |
| Education (*N*) | | | | | | |
|  | Graduate degree | 14 | 10 | 18 | 11 | 12 |
|  | Undergraduate degree | 19 | 17 | 12 | 19 | 18 |
|  | Secondary education | 2 | 1 | 5 | 2 | 6 |
|  | High school diploma | 13 | 7 | 9 | 8 | 5 |
|  | Technical/community college degree | 2 | 10 | 6 | 9 | 8 |

*Procedure.* After the main card game task, participants were asked to complete an intolerance of uncertainty (IU) questionnaire for exploratory purposes. Results for the exploratory analysis with this questionnaire is described at the end of the supplementary material section. Participants responded to the IU questionnaire only in Experiment 1A and 1B. In Experiment 1A, after the main task, participants were provided with two open-ended questions asking why they did or did not reveal cards early (the results of these questions were not reported in the current study). In the subsequent experiments we did not include the open-ended questions.

*Data analyses.* The main purpose of the study was to examine the difference between the predicted and actual card-revealing behaviors. We used the predicted card-revealing percentage and actual card-revealing percentage for each block for the following analyses, and call this within-subjects factor “Prediction” (predicted percentage vs. actual percentage). We analyzed the data using a repeated measures 2 x 2 x 2 x 3 ANOVA including Prediction (predicted and actual percentage), Cost (0, 10, and 20 points), Reward (50 and 100 points), and Win probability (25%, 50%, and 75%). Our main hypothesis is that there is a main effect of prediction, in the direction of actual card-revealing percentage higher than the predicted card-revealing percentage (i.e., participants underestimate the effect of curiosity on their tendency to reveal the card).

Two participants inaccurately answered both comprehension check questions (asking whether choosing to reveal the card would end the task early or not), and an additional 3 participants inaccurately answered one of the questions. Excluding these participants did not change the findings in terms of statistical significance reported below.

**Results**

Figure 1 summarizes the results. Although the pattern appears complicated, we can see a general trend where predicted frequency is lower than actual frequency, which is consistent with the main hypothesis. In line with the observation, a main effect of Prediction was significant *F* (1, 49) = 4.356, *p* = .042, η_p_^2^ = .082 suggesting that participants underestimated their card revealing in general.


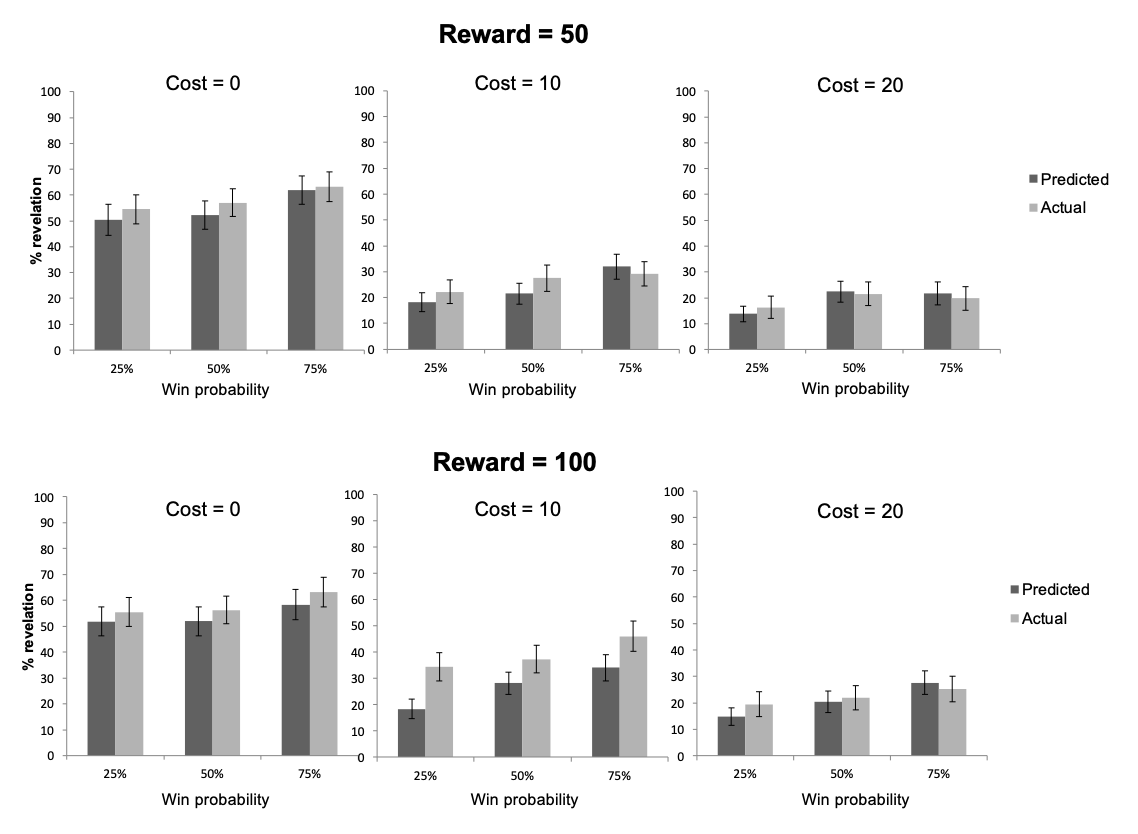


Figure 1. The prediction and actual card-revealing behavior in percentage as a function of reward, win probability, and cost level in Experiment 1A.

There was also a significant interaction of Prediction x Cost x Reward *F* (2, 98) = 4.141, *p* = .019, η_p_^2^ = .078 as well as Prediction x Reward *F* (1, 49) = 8.430, *p* = .006, η_p_^2^ = .147 and Prediction x Cost *F* (2, 98) = 5.123, *p* = .008, η_p_^2^ = .095. Other significant effects included: Cost *F* (2, 98) = 49.972, *p* < .001, η_p_^2^ = .505; Reward *F* (1, 49) = 5.634 *p* = .022, η_p_^2^ = .103; Win probability *F* (2, 98) = 11.508, *p* < .001, η_p_^2^ = .095; Cost x Reward *F* (2, 98) = 3.711, *p* = .028, η_p_^2^ *= .*070. Those interaction effects related to Prediction indicate that the hypothesized underestimation effect is modified by other factors (e.g., expected reward).

Next, in order to further examine the significant 3-way interaction, we conducted 2 (Prediction: predicted and actual percentage) x 2 (Reward: 50 points vs. 100 points) ANOVA with Prediction as a between subject factor at each level of Cost. At the 0 or the 20 cost level, nothing was significant. By contrast, at the 10 cost level, an interaction of Prediction x Reward was significant *F* (1, 49) = 8.699, *p* = .005, η_p_^2^ = .151 as well as Prediction *F* (1, 49) = 8.945, *p* = .004, η_p_^2^ = .154 and a main effect of Reward *F* (1, 49) = 9.882, *p* = .003, η_p_^2^ = .168. At the 10 cost–100 reward level, participants revealed cards more frequently than they predicted *F* (1, 49) = 11.586 *p* = .001 whereas at the 10 cost – 50 reward level, this was not significant *F* (1, 49) = 1.277 *p* = .264.

**Experiment 1B**

**Method**

*Participants.* See Table 2 for detailed information about participants. Three additional participants’ data were collected but removed prior to data analysis based on their response to the attention check question.

*Procedure and the design.* The procedure and the design were identical to Experiment 1A, except for the instructions. Specifically, in Experiment 1A, the instructions about the game were introduced across several pages, and participants were not able to return to previous pages even if they wished to, whereas in Experiment 1B, we provided a summary of the important instructions in a single page at the end of the instruction phase. Therefore, we sought to help participants better comprehend the structure and rules of the game. Secondly, we provided feedback to the comprehension check questions so that participants have an accurate understanding of the task (i.e., “That is correct (incorrect)! Whether you reveal cards or not it does not change the duration of the task”). In Experiment 1A, participants had no way of knowing whether their responses were correct. Even if they responded correctly to the comprehension check questions, their ideas about the task duration might change while being engaged in the task. By providing feedback we therefore sought to reinforce their understanding if they responded correctly in the first place, and correct their understanding if they erred. The comprehension check question after the task was removed in Experiment 1B. Like Experiment 1A, IU was tested at the end of the study. The results are described at the end of the supplementary material section. In the subsequent experiments reported below, IU was not included.

*Data analyses.* The same 2 x 3 x 2 x 3 ANOVA, including Prediction (predicted and actual percentage), Cost (0, 10, and 20 points), Reward (50 and 100 points), and Win probability (25%, 50%, and 75%), was conducted. Note again that we primarily focused on the main effect of Prediction. Because all participants received feedback on the comprehension check questions (about the task duration), unlike Experiment 1A, we did not rerun the analyses excluding participants who initially responded inaccurately to the questions. This was the same in the subsequent experiments reported below.

**Results and discussion**


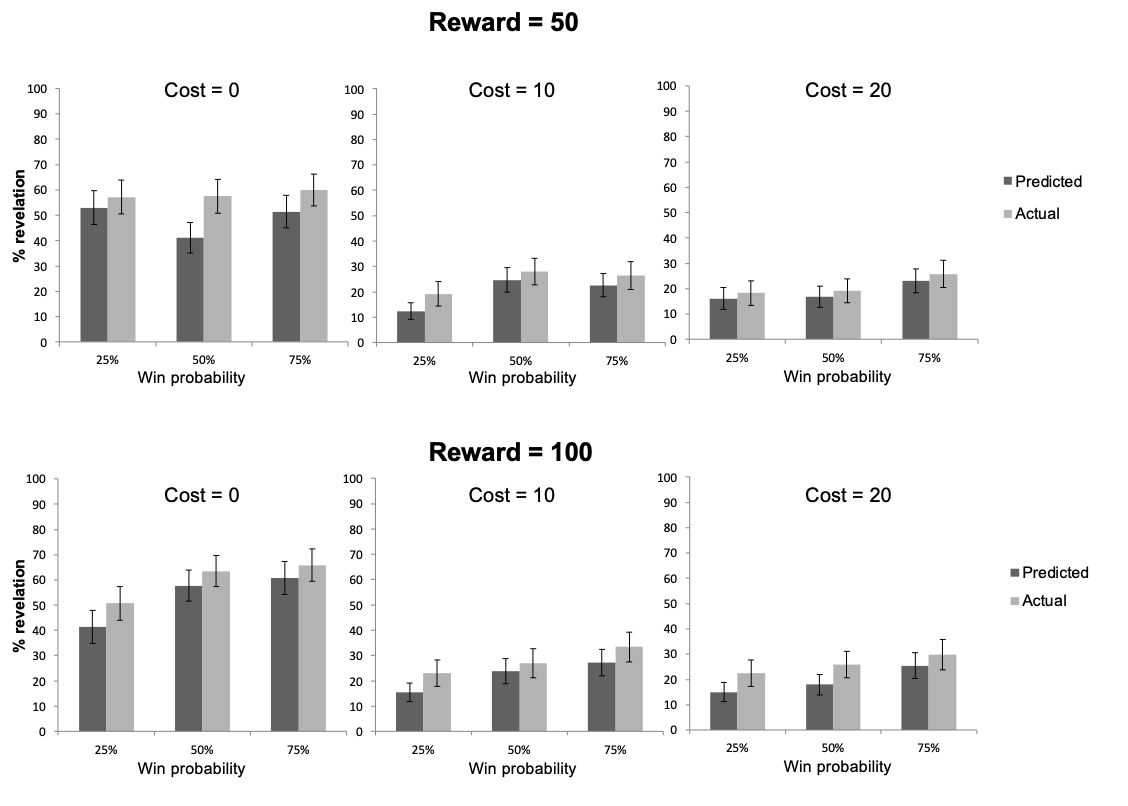


Figure 2. The prediction and actual card-revealing behavior in percentage as a function of reward, win probability, and cost level in Experiment 1B.

Figure 2 presents the percentage of all conditions. An inspection of Figure 2 shows again that overall participants underestimated their card-revealing behavior (i.e., they chose to reveal cards more frequently than predicted), consistent with the main hypothesis. Indeed, the main effect of Prediction was significant *F* (1, 44) = 4.435, *p* = .041, η_p_^2^ = .092, suggesting that participants revealed cards more frequently than they initially predicted.

There was also a significant interaction effect relating to Prediction, suggesting that the effects may be dependent on other factors. There was a significant 4-way interaction of Prediction x Cost x Win probability x Reward *F* (4, 176) = 2.718, *p* = .031, η_p_^2^ = .058, as well as a significant interaction of Prediction x Cost (2, 88) = 3.394, *p* = .038, η_p_^2^ = .072. Other significant effects unrelated to Prediction are the following: Cost *F* (2, 88) = 33.126, *p* < .001, η_p_^2^ = .430; Win probability *F* (2, 88) = 12.780, *p* < .001, η_p_^2^ = .225; Reward *F* (1, 44) = 4.861, *p* = .033. η_p_^2^ = .099; Cost x Reward x Win probability *F* (4, 176) = 3.735, *p* = .006, η_p_^2^ = .078.

To further understand the significant 4-way interaction, 2 (Prediction: predicted and actual percentage) x 2 (Reward: 50 points vs. 100 points) x 3 (Win probability: 25% vs. 50% vs. 75%) ANOVA with Prediction as a between subject factor was conducted at each level of Cost. At the 0 cost level, a 3-way interaction of Prediction x Reward x Win probability was significant *F* (2, 88) = 6.620, *p* = .002, η_p_^2^ = .131 as well as Prediction *F* (1, 44) = 8.109, *p* = .007. η_p_^2^ = .156 (there was a significant interaction of Reward x Win probability *F* (2, 88) = 5.558, *p* = .005. η_p_^2^ = .112 and a significant main effect of Win probability *F* (2, 88) = 3.586, *p* = .032. η_p_^2^ = .075). At the 0 cost–50 reward level, an interaction of Prediction x Win probability was significant, *F* (2, 88) = 4.058, *p* = .021. η_p_^2^ = .084 as well as a main effect of Prediction, *F* (1, 44) = 8.368, *p* = .006. η_p_^2^ = .160: at the 0 cost–50 reward–50% win probability and the 0 cost – 50 reward–75% win probability level, participants revealed cards more frequently than they predicted (75% win probability: *t* (44) = 2.459, *p* = .017; 50% win probability: *t* (44) = 3.377, *p* = .002 but not at the 0 cost–50 reward–25% win probability level *t* (44) = 1.018, *p* = .314. By contrast, at the 0 cost–100 reward level, a main effect of Prediction was significant, *F* (1, 44) = 5.219, *p* = .027. η_p_^2^ = .106 (and a main effect of Win probability *F* (2, 88) = 8.263, *p* = .001. η_p_^2^ = .158).

At the 10 cost level, only a main effect of Win probability was significant *F* (2, 88) = 6.046, *p* = .003. η_p_^2^ = .121. At the 20 cost level, an interaction of Prediction x Reward was significant *F* (1, 44) = 4.251, *p* = .045. η_p_^2^ = .088 as well as a main effect of Win probability *F* (2, 88) = 7.471, *p* = .001. η_p_^2^ = .145. At the 20 cost–100 reward level, participants revealed cards more frequently than they predicted, *F* (1, 44) = 8.368, *p* = .006. η_p_^2^ = .160 whereas at the 20 cost–50 reward level this was not significant *F* (1, 44) = .688, *p* = .411.

**Experiment 1C**

**Method**

*Participants.* See Table 2 for detailed information about participants. No participants were removed.

*Procedure and the design.* The procedure was identical to Experiment 1B, except for the elimination of the 20 cost level condition. As a result, there were a total of 12 blocks instead of 18, each of which consisted of 8 trials. We removed the 20 cost condition because participants rarely revealed the cards in this condition, and not much variability was present to detect the underestimation effect. As in Experiment 1B participants were given feedback on whether their answers to the questions about the task duration were correct. We did not include the IU questionnaire in Experiment 1C (nor in the subsequent experiments).

*Data analysis.* The 2 x 2 x 2 x 3 ANOVA, including Prediction (predicted and actual percentage), Cost (0, and 10 points), Reward (50 and 100 points), and Win probability (25%, 50%, and 75%), was conducted. Again, we expected the significant main effect of Prediction.

**Results**

Figure 3 presents the card-reveal frequency for each condition.


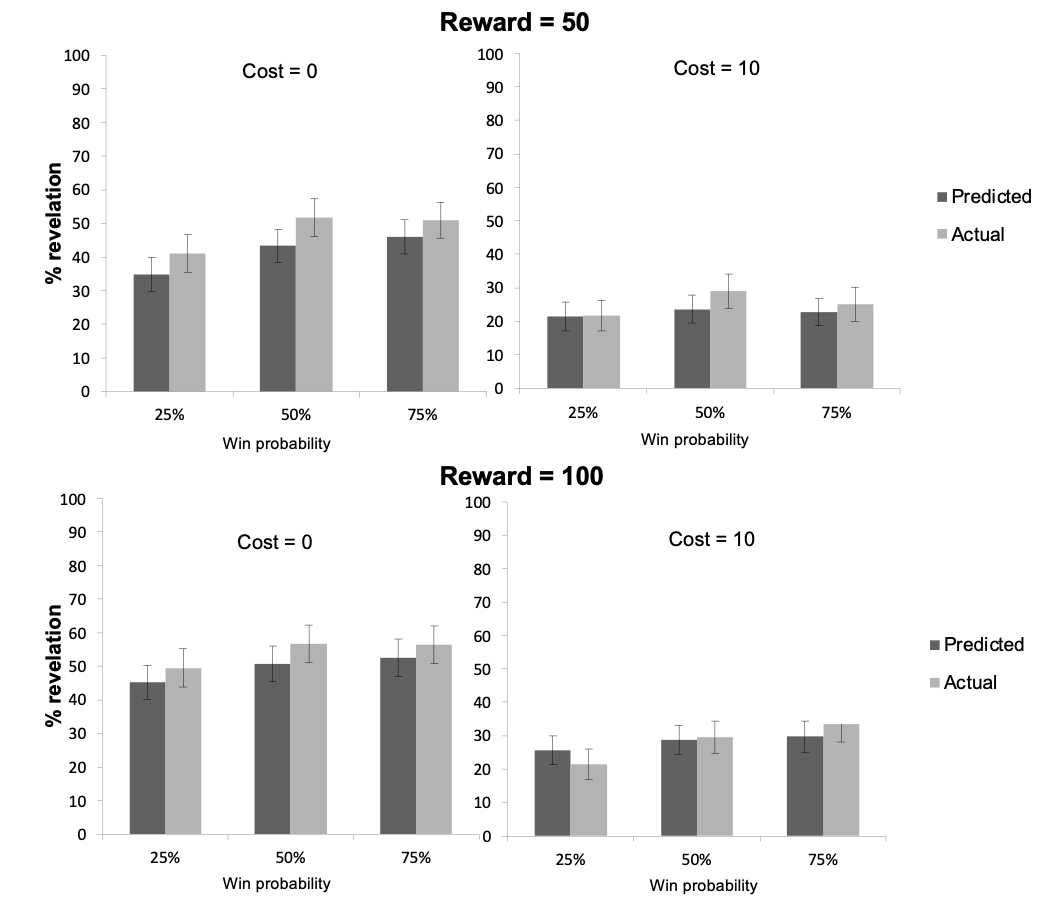


Figure 3. The prediction and actual card-revealing behavior in percentage as a function of reward, win probability, and cost level in Experiment 1C.

Again, an inspection of Figure 3 shows that participants seem to underestimate their information seeking. Unlike the previous experiments, however, the main effect of Prediction did not reach statistical significance, *F* (1, 49) = 2.642, *p* = .110. The only significant effect related to Prediction was a significant interaction of Prediction x

Cost *F* (1, 49) = 5.602, *p* = .022, η_p_^2^ = .103. Participants underestimated curiosity by revealing cards more frequently than they previously predicted at the 0 cost level, *F* (1, 49) = 6.373, *p* = .015. *η_p_^2^* = .115 whereas this was not significant at the 10 cost level *F* (1, 49) = .328, *p* = .569. Other significant effects included: Cost *F* (1, 49) = 27.610, *p* < .001. η_p_^2^ = .360; Reward *F* (1, 49) =12. 288, *p* = .001. η_p_^2^ = .200; Win probability *F* (1, 49) =6.492, *p* = .001. η_p_^2^ = .117.

**Experiment 1D**

**Method**

*Participants.* See Table 2 for detailed information about participants. An additional participant was tested but based on their response to the attention check question was excluded prior to data analysis.

*Procedure and the design.* We used the same design as in Experiment 1B. The current experiment was different from previous experiments in that participants could see the card identity (i.e., the card type and associated points) in each trial *only* when they paid some cost. If they did not pay a cost then the outcome was not displayed at the end of the trial (i.e., after 5 seconds) (“Your game reward points and total reward points so far will appear on the screen – but only when you choose to pay some costs. When the cost is 0, even if you do not choose to reveal cards, the card identity and total points earned will be displayed. When the cost is -10 or -20, if you do not choose to reveal cards, the card identity and total points earned will NOT be displayed”). Irrespective of their decision to pay a cost, however, the total earned points were still calculated behind the scene and displayed at the end of each block. Like previous experiments, information request did not have any effects on their points obtained, but this modified procedure made the delivery of the information significantly delayed. By doing so we sought to enhance the level of card-revealing behavior after observing that in previous experiments, the overall tendency to reveal cards was quite low (about 36.28% ~ 38.90%).

*Data analysis.* Like Experiment 1A, we analyzed the data using a repeated measures 2 x 3 x 2 x 3 ANOVA including Prediction (predicted and actual percentage), Cost (0, 10, and 20 points), Reward (50 and 100 points), and Win probability (25%, 50%, and 75%). Again, we expected the main effect of Prediction, hypothesizing that participants’ actual percentage is significantly higher than predicted percentage (underestimation).

**Results and discussion**

Figure 4 presents the descriptive statistics of each condition. Overall, the percentage of card-revealing behavior increased in comparison to the previous experiments (Revealing in Experiment 1D: 48.26 % on average whereas 37.33 % in Experiment 1A, 36.28%, Experiment 1B, and 38.91% in Experiment 1C). Again, we can see the tendency that participants underestimated the information seeking frequency.


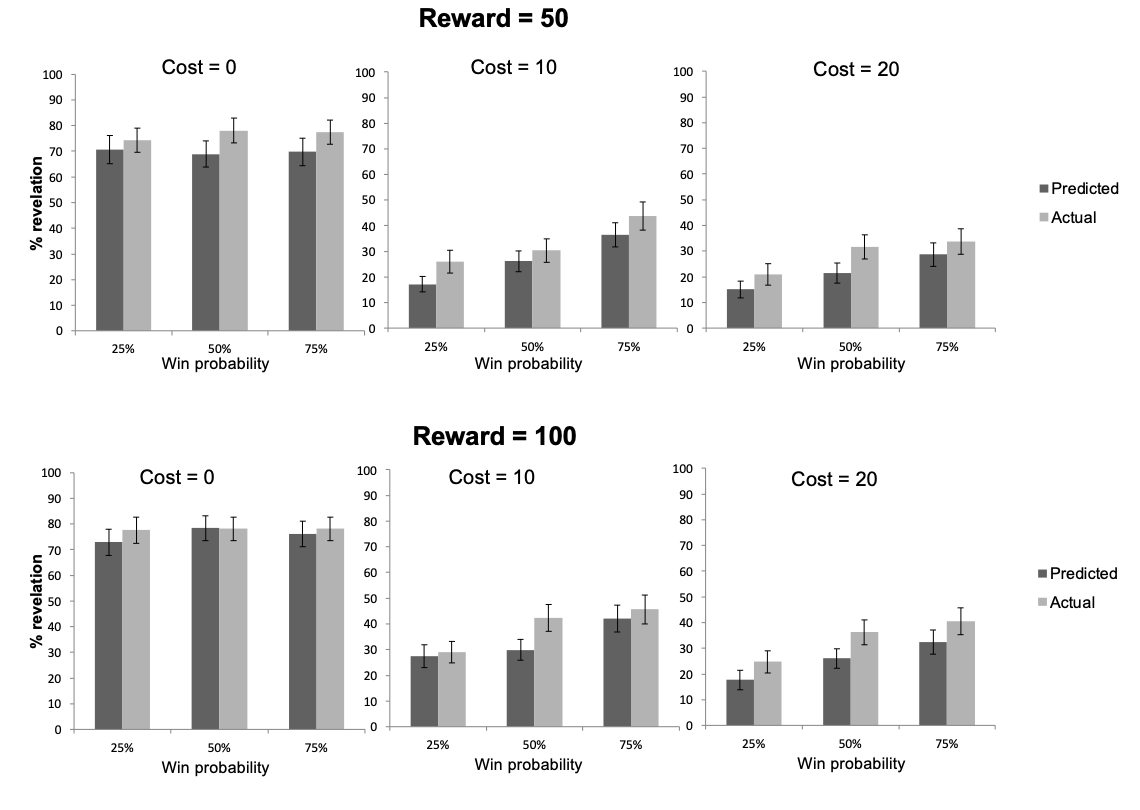


Figure 4. The prediction and actual card-revealing behavior in percentage as a function of reward, win probability, and cost level in Experiment 1D.

Consistent with our hypothesis, the main effect of Prediction was significant *F* (1, 48) =27.841, *p* < .001, η_p_^2^ = .367 suggesting participants underestimated the effects of curiosity on card-revealing behavior.

The 4-way interaction of Prediction x Cost x Reward x Win was also significant *F* (4, 192) = 3.696, *p* = .006, η_p_^2^ = .071, indicating that the effect is dependent on other factors. To further understand the 4-way interaction effect, 3-way ANOVA was conducted at each cost level. At the 0 cost level, an interaction of Prediction x Reward was significant *F* (1, 48) = 5.223, *p* = .027, *η_p_^2^* = .098 as well as a main effect of Prediction *F* (1, 48) = 5.193, *p* = .027, *η_p_^2^* = .098: participants underestimated curiosity in the 50 reward condition *F* (1, 48) = 8.831, *p* = .005, *η_p_^2^* = .155 whereas this was not significant in the 100 reward condition *F* (1, 48) = .975, *p* = .328. At the 10 cost level, an interaction of Prediction x Reward x Win was significant *F* (2, 96) = 5.561, *p* = .005, *η_p_^2^* = .104 as well as a main effect of Prediction *F* (1, 48) = 12.432, *p* = .001, *η_p_^2^* = .206.^[[1]](#footnote-1)^ Follow-up tests showed that a main effect of Prediction was significant at the 10 cost level–50 reward level *F* (1, 48) = 11.266, *p* = .002, *η_p_^2^* = .190. An interaction of Prediction x Win probability was not significant (*p* = .353).^[[2]](#footnote-2)^ There was a significant interaction of Prediction x Win probability *F* (2, 96) = 5.408, *p* = .006, *η_p_^2^* = .101 as well as a main effect of Prediction at the 10 cost –100 reward level *F* (1, 48) = 8.630, *p* = .005, *η_p_^2^* = .152.^[[3]](#footnote-3)^ Participants’ underestimation of curiosity was observed only in the 50% win probability condition at 10 cost –100 reward level *F* (1, 48) = 17.342, *p* < .001, *η_p_^2^* = .265 (other win probability conditions: *p* values > .05). Finally, at the 20 cost level, a main effect of Prediction was significant *F* (1, 48) = 22.243, *p* < .001, *η_p_^2^* = .317: participants underestimated curiosity.^[[4]](#footnote-4)^

Other significant effects unrelated to Prediction included: Cost x Win probability *F* (4, 192) = 5.522, *p* < .001, η_p_^2^ = .103; Win probability *F* (2, 96) = 22.059, *p* < .001, η_p_^2^ = .315; Reward *F* (1, 48) = 13.970, *p* < .001, η_p_^2^ = .225; Cost *F* (2, 96) = 99.904, *p* < .001, η_p_^2^ = .675.

**Experiment 1E**

**Method**

*Participants.* See Table 2 for detailed information about participants. No participants were excluded based on the attention check question criteria.

*Procedure and the design.* The same method as in Experiment 1D was used except that at the beginning of the task participants were asked to imagine someone who is similar to themselves (“Imagine someone who is similar to yourself. Let’s call her/him Jane/Mike”) and make predictions about his or her card revealing in each block (“How frequently do you think Jane/Mike will reveal the cards?”). Then participants engaged in the same card game as in Experiment 1D. By doing so we aimed to test the robustness of the hypothesis using a slightly different method for metacognition. We still predicted that participants would display an underestimation of others’ curiosity (in comparison to their own card-revealing tendency) (see Murayama et al., 2016).

*Data analysis.* Like previous experiments, we analyzed the data using a repeated measures 2 x 3 x 2 x 3 ANOVA including Prediction (predicted and actual percentage), Cost (0, 10, and 20 points), Reward (50 and 100 points), and Win probability (25%, 50%, and 75%). Again, we expected the main effect of Prediction, hypothesizing that participants’ actual percentage is significantly higher than predicted percentage (underestimation).

**Results**

Figure 5 presents the results. Once again, the pattern seems to be consistent with the main hypothesis that people underestimate the effect of curiosity on information-seeking behavior.


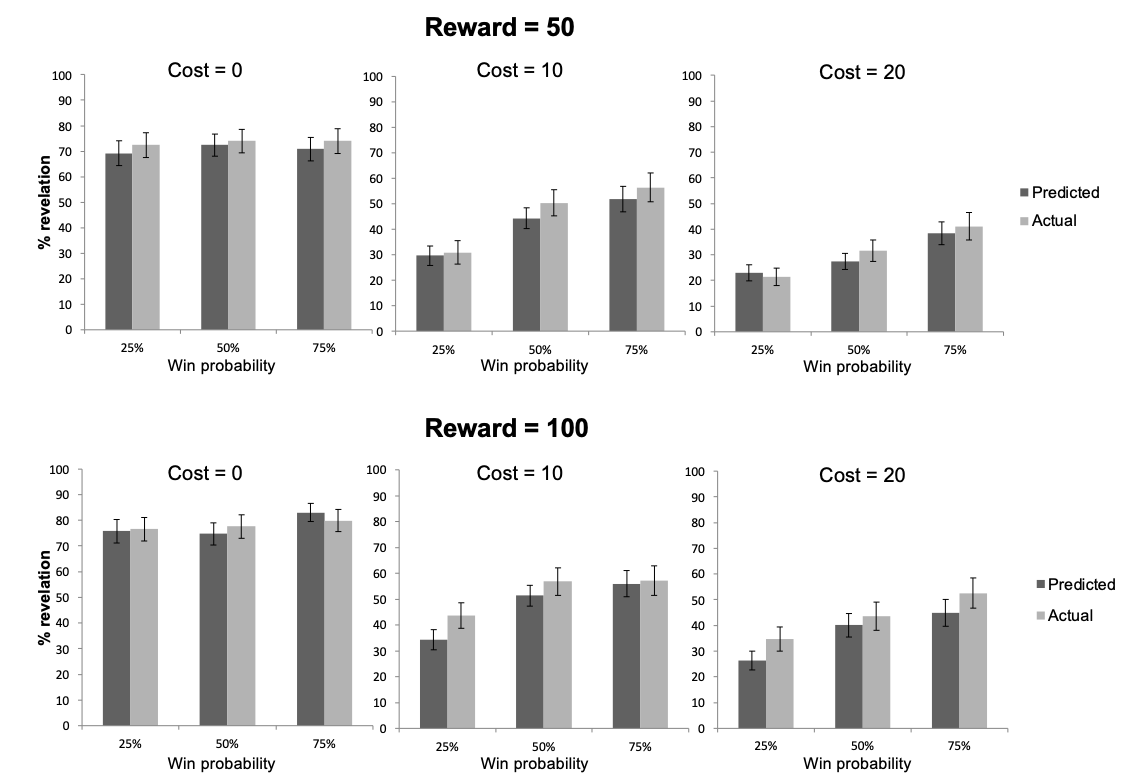


Figure 5. The third-party prediction and own card-revealing behavior in percentage as a function of reward, win probability, and cost level in Experiment 1E.

Consistent with the observation, the analysis revealed a significant main effect of Prediction *F* (1, 48) = 5.871, *p* = .019, η_p_^2^ = .109, showing that participants had a higher frequency of information-seeking behavior than they had predicted. There were no other significant interaction effects related to Prediction. Other significant effects unrelated to Prediction were: Cost *F* (2, 96) = 84.470, *p* < .001, η_p_^2^ = .638; Reward *F* (1, 48) = 26.079, *p* < .001, η_p_^2^ = .352; Win probability *F* (2, 96) = 25.522, *p* < .001, η_p_^2^ = .347; Cost x Win probability *F* (4, 192) = 7.506, *p* < .001, η_p_^2^ = .135.

**Random effects of experiments**

We tested the statistical significance of random effects (slopes) of participants and experiments using log-likelihood test, and the results are indicated in Table 1. The random effect of experiments is of particular interest, as the presence of this random effect indicates that the results are statistically different across experiments, likely caused by some design difference. The only significant random effect of experiment related to Prediction was Prediction x Cost x Reward, variance estimate = 8.604, χ^2^(1) = 17.4, *p* < .001. To understand how this interaction effect is different across experiments, we first computed the difference between predicted and actual percentages (i.e., the extent of overestimation) and then further computed the difference between Reward 100 and 50 conditions (i.e., a positive value means that Reward 100 condition has a bigger overestimation). The plots of these “difference of difference” scores (which is equivalent to the 2-way Prediction x Reward interaction) are displayed in Figure 6. As can be seen, this 2-way interaction seems to be similar between Cost 0 and Cost 10 conditions except for Experiment 1A, which showed the underestimation effect particularly when both cost and reward are higher (10 and 100). This observation is consistent with the finding that Prediction x Cost x Reward interaction was significant only in Experiment 1A. The only thing that differentiates Experiment 1A from all of the other experiments (1B-1E) is the change of the instructions – we improved the instructions and feedback so that participants could correctly grasp the structure of the task and understand that skipping the information-seeking behavior would not shorten the total experimental time. While it is not easy to make a definite interpretation about the findings, we speculate that participants in Experiment 1A showed a particularly bigger underestimation effect when cost and reward are both higher perhaps because of the absence of a full summary about the game structure (provided at the end of the instruction in experiments 1B-1E). This might have made it difficult for participants to gauge the value of cost; as a result, participants might have overestimated the value of high cost, especially when the reward is high stake.


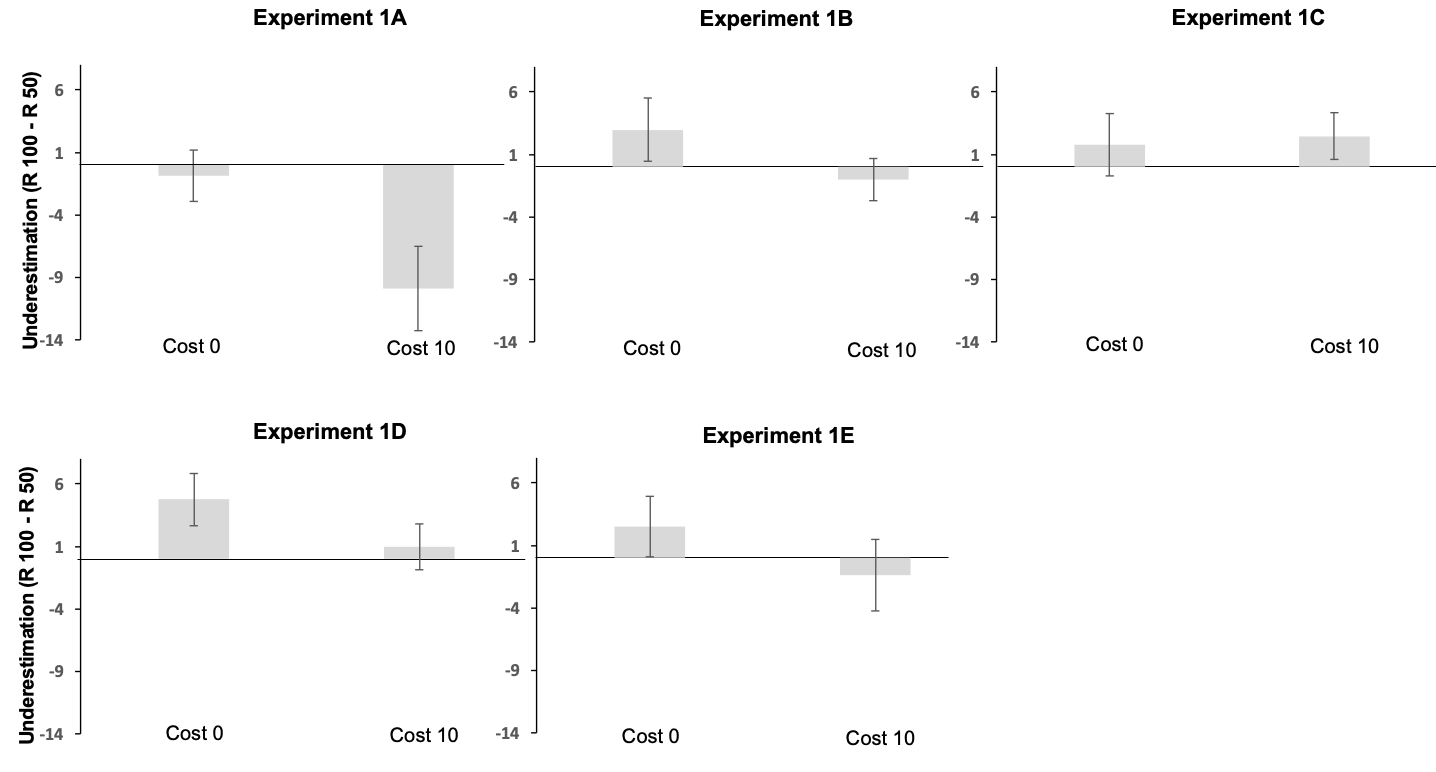


Figure 6. The difference scores (predicted – actual revealing) was compared between Reward 100 and Reward 50 conditions.

**Comparison between Experiment 1B & 1C.**

The weakest underestimation effect was obtained in Experiment 1C (mean % difference = -3.52, *p* = .110), raising the possibility that the inclusion of Cost 20 condition, which was removed only in Experiment 1C, contributed to the observed findings. To test this idea, we compared the results between Experiment 1B (which was most similar to Experiment 1C) and Experiment 1C by conducting a 2 (Prediction: predicted and actual revealing) x 2 (Experiment: Experiment 1B and Experiment 1C) ANOVA, excluding Cost 20 condition. The interaction between Prediction and Experiment was not statistically significant, *F*(1, 93) = .796, *p* = .374, indicating that the observed difference in the statistical significance of the effects of prediction can be reasonably explained by sampling error. Nevertheless, we reserve the possibility that the presence of a large cost condition (Cost 20 condition) strengthened the underestimation effect, perhaps because participants might have felt that -10 points were relatively smaller than -20 points and have engaged in information-seeking more frequently. In fact, participants’ underestimation was statistically significant in the Cost 0 but not in the 10 Cost 10 condition in Experiment 1C.

**Experiment 2**

**Testing the impact of the prediction on non-instrumental information-seeking**

*Participants.* The participants were drawn from the same population as in previous experiments. Sixteen participants requested to have their data removed and were subsequently excluded, resulting in a total sample size of 336 participants (194 males). The mean age of the participants was 40.02 years (SD = 12.11). The majority of the participants identified as White (N = 264), followed by Black (N = 24), Asian (N = 23), and Other/Mixed (N = 20). Five participants did not provide their racial information. Due to an experimenter error, only 70 participants' educational attainment data were collected. Among these, the majority had either an undergraduate degree (N = 24) or a graduate degree (N = 22). A smaller number of participants reported having completed secondary education (N = 5), a high school diploma (N = 8), or attended technical/community college (N = 11).

**Attention check questions asked at the end of the study**

We included attention check questions at the end of the study to assess whether participants paid attention to the task in the study. First, participants were told that, “We intend to use these data for research purposes. If you did not pay attention or otherwise provided useless data, it would be helpful for us to know this so that we do not include your responses in our research. Should we eliminate your data for this reason? Yes/no.” We excluded participants based on their responses in all the experiments: Experiment 1A (*N* = 1), Experiment 1B (*N* = 3), Experiment 1C (*N* = 0), Experiment 1D (*N* = 1), Experiment 1E (*N* = 0) and Experiment 2 (*N* = 16). We also asked participants a second question: “In this experiment, did you concentrate on the tasks? Please rate from 1 (not at all) to 5 (very much).” We did not use their responses to this question – the decision was made a priori but we confirm that their response to the second question is consistent with their response to the first question (e.g., no participants asked to remove their data and rated their concentration level as high). Participants were then asked a final question: “What do you think the study was about?” We do not report their response in this manuscript. However, we confirm that no participants were aware of the aim of the study.

**IU questionnaire exploratory analyses**

IU has two subscales: The first subscale relates to a belief that uncertainty has negative implications for self (e.g., “Uncertainty stops me from having a firm opinion.”) while the second subscale relates to a belief that uncertainty is unfair and negatively affects life (e.g., *“*It frustrates me not having all the information I need”) (Buhr & Dugas, 2002; Sexton & Dugas, 2009). Participants’ discrepancy scores (prediction – actual percentage of revealing cards) were significantly related to a belief about an unfair aspect of uncertainty (*r* = .303, *p* = .032) but not to a belief about negative self consequence of uncertainty (*r* = .128, *p* = .375) in Experiment 1A. That is, the more participants thought that uncertainty is unfair and ruins life, the more they accurately predicted their revealing. This might suggest that a certain negative belief about uncertainty leads them to be aware of their tendency or willingness to eliminate uncertainty (in this case, by revealing information earlier). No significant relations related to the discrepancy scores were found in Experiment 1B, however: a belief about an unfair aspect of uncertainty (*r* = -.105, *p* =. 492); a belief about negative self consequence of uncertainty (*r* = -.172, *p* = .259). See Figure 7. In both experiments these two factors were related to each other (Experiment 1A: *r =* .74; Experiment 1B: *r* = .76, both *p* values less than .001).


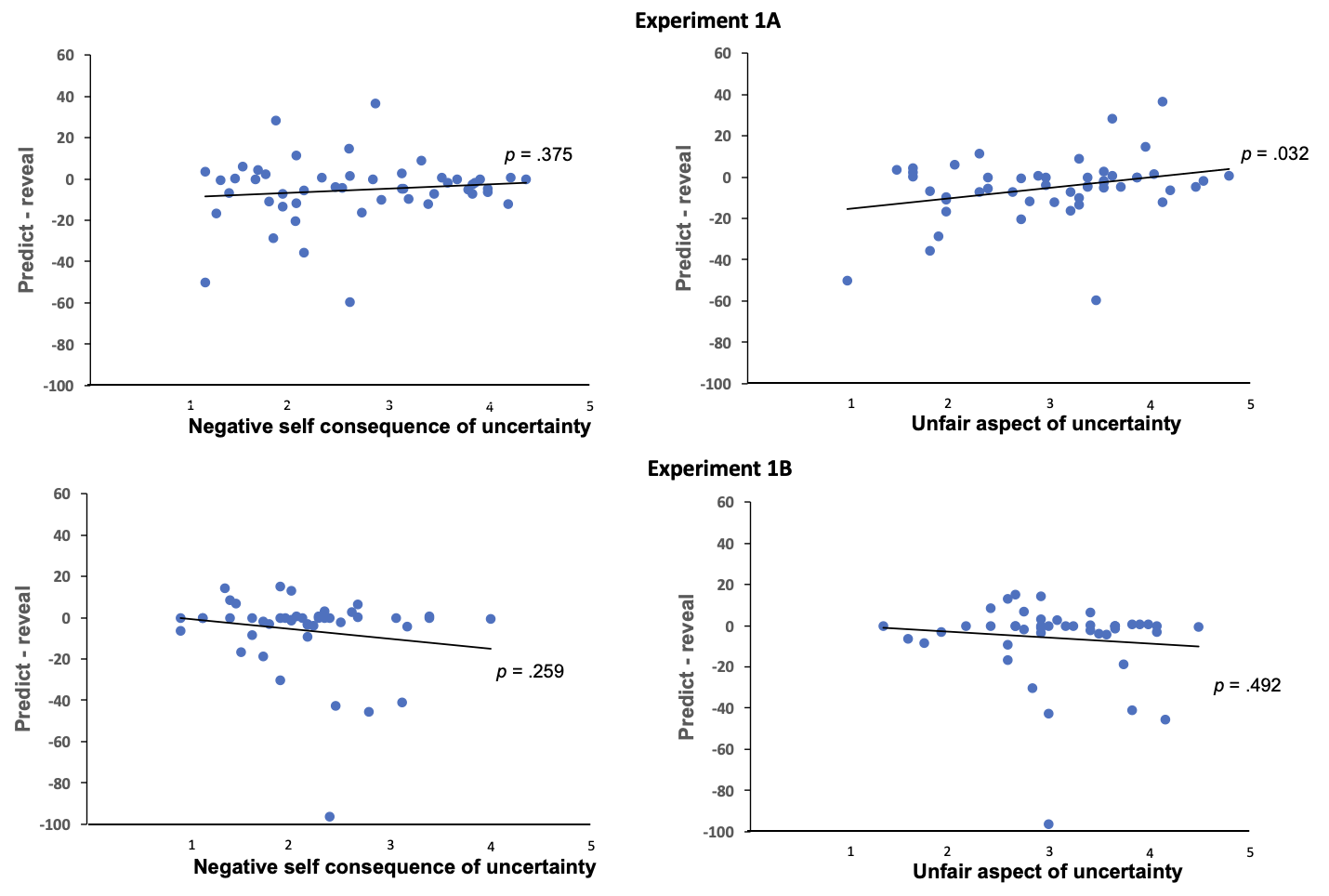


Figure 7. Scatter plots depicting a relationship between Intolerance of Uncertainty and discrepancy (predict – reveal) in Experiment 1A and 1B.

**Instruction about the task duration**

Participants were explicitly instructed that the entire task duration does not change regardless of whether they reveal cards: “Revealing the card early will not affect the card identity. Revealing the card early will not affect the total duration of the task, either. Regardless of whether you reveal the card early, the interval between the start of a trial and the start of the next trial will remain the same.”

1. Both main effects of Reward and Win probability were significant: Reward *F* (1, 48) = 14.800, *p* < .001, η_p_^2^ = .236; Win probability *F* (2, 96) = 18.590, *p* < .001, η_p_^2^ = .279. [↑](#footnote-ref-1)
2. A main effect of Win probability was significant *F* (2, 96) = 20.343, *p* < .001, η_p_^2^ = .298. [↑](#footnote-ref-2)
3. A main effect of Win probability was significant *F* (2, 96) = 9.758, *p* < .001, η_p_^2^ = .169. [↑](#footnote-ref-3)
4. Both main effects of Win probability and Reward were significant: Win probability *F* (2, 96) = 18.739, *p* < .001, η_p_^2^  = .281; Reward *F* (1, 48) = 6.141, *p* = .017, η_p_^2^ = .113. [↑](#footnote-ref-4)
